# Understanding of Molecular Pain Medicine: Genetic Basis of Variation in Pain Sensation and Analgesia Response

**DOI:** 10.5812/aapm.8583

**Published:** 2013-01-01

**Authors:** Mahsa Motavaf, Saeid Safari, Seyed Moayed Alavian

**Affiliations:** 1 Department of Molecular Medicine, Rezvan Medical Research Institute, Tehran, Iran; 2 Department of Anesthesiology, Rasoul Akram Medical Center, Iran University of Medical Sciences (IUMS), Tehran, Iran; 3 Baqiyatallah Research Center for Gastroenterology and Liver Diseases, Baqiyatallah University of Medical Sciences, Tehran, Iran

**Keywords:** Analgesia, Genetic Association Studies, Pain, Single Nucleotide Polymorphism

Pain is a fundamental experience with sensory, emotional and cognitive aspects. Though pain is often a normal part of the human condition, in the clinical setting, there is vast inter-individual variability in the severity of pain reported by patients with apparently similar pain states, as well as widely differing analgesic dosing requirements to produce excellent pain relief with tolerable side-effects. This variability in pain sensitivity and the response to analgesic manipulations remains a considerable clinical challenge as well as an area of intense scientific investigation (1, 2). This inter-individual variability is a result of genetic factors, environmental factors, as well as the complex interaction of the two. Genetic variation has been shown to explain a significant portion of this variability. However, the fact is that studies of genetic differences in pain-related traits have been largely neglected. Heritability estimates based on inbred strains of laboratory mice studies suggest that genetic factors explain up to 30 to 76% of the variance in pain responding (3, 4). Twin studies suggest that genetic factors contribute to the differences in individuals’ pain-related phenotypes with heritability estimates of up to 70% for clinical pain conditions and up to 60% for sensitivity to certain kinds of experimental stimuli (5-7). The identification of genetic variations affecting propensity to pain raises the possibility of discovering new therapeutic targets for pain. To date a large number of candidate ‘pain’ genes have been identified as potentially contributing to heritable differences in pain sensitivity and analgesic responsiveness in animals and humans. Since then, many genetic association studies have been conducted in humans to investigate the possibility that single nucleotide polymorphisms (SNPs) in an individual gene that may explain these differences. Single functional SNPs or combinations of SNP alleles that are generally tend to be inherited together (haplotypes) can contribute to increased or decreased susceptibility to pain. SNPs in more than 20 genes that affect pain sensitivity or analgesic responses have been identified in the human genome. These are summarized in Table. However, the study of pain-related SNPs in the human yield conflicting conclusions; in many cases, a finding from one has been contradicted by those from others. One example of these genes is Catechol-O-methyl Transferase (COMT). COMT is involved in the inactivation of the endogenous catecholamine neurotransmitters, playing key roles in the physiological modulation of analgesia and pain processing. It has been estimated that variations in the gene coding for COMT explain approximately 10% of variability in pain sensitivity (8). A functional valine-to-methionine SNP at position 158 in COMT gene (called rs4680) has been proposed to contribute significantly increased experimental pain responses (9). The form of the enzyme containing methionine is much less active in the brain than the one containing valine. The enzyme with increased activity has higher ability to activate μ-opioid neurotransmission in basal ganglia, thalamus, limbic and paralimbic areas in response to a sustained pain challenge resulting in decreased pain sensation (10). The mu-opioid receptor gene (OPRM1) A118G polymorphism is another promising candidate for both opioid response and pain sensitivity because of both pain-related biological functions and apparent experimental and clinical evidence. A118G SNP of the OPRM1 gene is demonstrated to be associated with responses to three different experimental pain modalities, including thermal, mechanical, and ischemic pain (11). Experimental short duration heat also shows the effects of TRPA1 variations on pain sensitivity (12). Sensitivity to analgesics, including gabapentin, morphine, and NSAIDS, is also affected by genetic factors (13). For example, polymorphisms of the cytochrome P450 enzymes (CYP), which play a key role in the metabolism of many drugs, can affect the efficacy of opiates and NSAIDs. Depending on the activity of the metabolites compared to the original drug, reduced activity of cytochromes can either reduce or enhance analgesic efficacy. For example, O-desmethyltramadol, which is one metabolite of the opioid tramadol, is a considerably more potent agonist of the µ-opioid receptor than tramadol. This means that reduced metabolization of tramadol display reduced analgesia, despite an increased half-life of tramadol being observed. Polymorphisms in the gene coding for cytochrome P450 2D6 (CYP2D6), which reduces its activity, is associate with reduced tramadol metabolism and effectiveness of this analgesic (8). Furthermore, the variations in the melanocortin-1 receptor gene have been shown to affect µ-opioid analgesia in both mice and humans (9). It is demonstrated that polymorphisms affecting the activity of the multidrug resistance protein ABCB1 (MDR1), which is a major determinant of morphine bioavailability, can alter the efficacy of morphine pain relief (10). By affecting the rate of morphine and its metabolites removal from the cell this polymorphism alters the efficacy this analgesic. The control of pain has been a major goal of pharmacotherapy from the earliest times. Inter-individual variability in pain sensitivity and analgesic drug responsiveness in the clinical setting appears to be underpinned by complex interactions between multiple genetic and environmental factors (14). Identification of genetical variations of alleles that affect the pharmacokinetics or pharmacodynamics of medications used for pain management gives the opportunity to the physicians to select the appropriate analgesic drug and dosing regimen for an individual patient, instead of empirical selection and dosing escalation (15, 16). These data emphasize the need to understand how genes influence individuals’ response to pain and analgesic drug. In addition to its basic intellectual values, understanding the influence of genetic variation on pain sensitivity, variable responses is also highly clinically valuable as it may lead to more individualized care for patients and the identification of novel therapeutic targets. In the near future, pharmacogenomic approaches offer the field of pain medicine the opportunity to gain new insights into the pathophysiology of differences in individuals’ analgesic responses and pain sensitivity (17). If we open and collaborative approach to performing the relatively large studies required for reliable genetic analysis, this approach may provide new insights into pain treatments.

**Table.** SNPs Suggested to Affect Human Pain Sensitivity and Analgesic Response

Gene	Protein	Mutation	Phenotype	Example Refrences
**LTA**	Lymphotoxin-alpha	Multiple SNPs	Altered pain sensitivity	(18)
**PTGS2**	Prostaglandin-endoperoxide synthase 2	Multiple SNPs	Altered pain sensitivity	(18)
**SCN9A**	Sodium channel, voltage-gated, type IX, alpha subunit.	SNP	Increased pain	(19, 20)
**COMT**	Catechol-O-methyltransferase	Multiple SNPs	Increased/decreased pain sensitivity	(12, 21)
**GCH1**	GTP cyclohydrolase	Multiple SNPs	Partial analgesia	(22)
**OPRM1**	Opioid receptor m1	Multiple SNPs	Decreased pain sensitivity, decreased opioid analgesia	(11)
**OPRD1**	Opioid receptor d1	Multiple SNPs	Increased/decreased pain sensitivity	(23)
**MC1R**	Melanocortin 1 receptor	Loss of function SNPs?	Partial analgesia, increased analgesic responsiveness	(24)
**TRPA1**	Transient receptor potential A1	Multiple SNPs	Increased pain sensitivity	(12)
**TRPV1**	Transient receptor potential V1	SNP	Decreased pain sensitivity	(23)
**CYP2D6**	Cytochrome P450 2D6	Multiple SNPs	Altered analgesic efficacy	(8)
**ABCB1**	ATP-binding cassette, B1	SNP	Altered morphine sensitivity	(10)
**FAAH**	Fatty acid amide hydrolase	Multiple SNPs	Increased pain sensitivity	(12)

## References

[1] Farzanegan G, Alghasi M, Safari S (2011). Quality-of-Life Evaluation of Patients Undergoing Lumbar Discectomy using Short Form 36.. Anesth Pain..

[2] Hajiesmaeili M, Safari S (2012). Pain Management in Intensive Care Unit: Do We Need Special Protocols?. Anesth Pain..

[3] Lariviere WR, Wilson SG, Laughlin TM, Kokayeff A, West EE, Adhikari SM (2002). Heritability of nociception. III. Genetic relationships among commonly used assays of nociception and hypersensitivity.. Pain..

[4] Mogil JS, Wilson SG, Bon K, Eun Lee S, Chung K, Raber P (1999). Heritability of nociception II. Types' of nociception revealed by genetic correlation analysis.. Pain..

[5] Lacroix-Fralish ML, Mogil JS (2009). Progress in genetic studies of pain and analgesia.. Annu Rev Pharmacol..

[6] Nielsen C, Knudsen G, Steingrimsdottir O (2012). Twin studies of pain.. Clin Genet..

[7] Ritter C, Bingel U (2009). Neuroimaging the genomics of pain processing--a perspective.. Neuroscience..

[8] Stamer UM, Stuber F (2007). Codeine and tramadol analgesic efficacy and respiratory effects are influenced by CYP2D6 genotype.. Anaesthesia..

[9] Mogil JS, Ritchie J, Smith SB, Strasburg K, Kaplan L, Wallace MR (2005). Melanocortin-1 receptor gene variants affect pain and mu-opioid analgesia in mice and humans.. J Med genet..

[10] Campa D, Gioia A, Tomei A, Poli P, Barale R (2008). Association of ABCB1/MDR1 and OPRM1 gene polymorphisms with morphine pain relief.. Clin pharma therap..

[11] Fillingim RB, Kaplan L, Staud R, Ness TJ, Glover TL, Campbell CM (2005). The A118G single nucleotide polymorphism of the mu-opioid receptor gene (OPRM1) is associated with pressure pain sensitivity in humans.. J Pain..

[12] Kim H, Mittal DP, Iadarola MJ, Dionne RA (2006). Genetic predictors for acute experimental cold and heat pain sensitivity in humans.. J Med Genet..

[13] Wilson SG, Bryant CD, Lariviere WR, Olsen MS, Giles BE, Chesler EJ (2003). The heritability of antinociception II: pharmacogenetic mediation of three over-the-counter analgesics in mice.. J Pharmacol Exp Ther..

[14] Imani F, Safari S (2011). “Pain Relief is an Essential Human Right”, We Should be Concerned about It.. Anesth Pain..

[15] Jannetto P, Bratanow N (2010). Pharmacogenomic considerations in the opioid management of pain.. Genome Med..

[16] Shoar S, Esmaeili S, Safari S (2012). Pain Management After Surgery: A Brief Review.. Anesth Pain..

[17] Kim H, Clark D, Dionne RA (2009). Genetic contributions to clinical pain and analgesia: avoiding pitfalls in genetic research.. J Pain..

[18] Rausch SM, Gonzalez BD, Clark MM, Patten C, Felten S, Liu H (2012). SNPs in PTGS2 and LTA predict pain and quality of life in long term lung cancer survivors.. Lung cancer..

[19] Reimann F, Cox JJ, Belfer I, Diatchenko L, Zaykin DV, McHale DP (2010). Pain perception is altered by a nucleotide polymorphism in SCN9A.. P Natl Acad Sci USA..

[20] Greenbaum L, Tegeder I, Barhum Y, Melamed E, Roditi Y, Djaldetti R (2012). Contribution of genetic variants to pain susceptibility in Parkinson disease.. Eur J Pain..

[21] Diatchenko L, Slade GD, Nackley AG, Bhalang K, Sigurdsson A, Belfer I (2005). Genetic basis for individual variations in pain perception and the development of a chronic pain condition.. Hum Mol Genet..

[22] Tegeder I, Costigan M, Griffin RS, Abele A, Belfer I, Schmidt H (2006). GTP cyclohydrolase and tetrahydrobiopterin regulate pain sensitivity and persistence.. Nature Med..

[23] Kim H, Neubert JK, San Miguel A, Xu K, Krishnaraju RK, Iadarola MJ (2004). Genetic influence on variability in human acute experimental pain sensitivity associated with gender, ethnicity and psychological temperament.. Pain..

[24] Mogil JS, Wilson SG, Chesler EJ, Rankin AL, Nemmani KV, Lariviere WR (2003). The melanocortin-1 receptor gene mediates female-specific mechanisms of analgesia in mice and humans.. P Natl Acad Sci USA..

